# Crisis and chaos in behavioral nutrition and physical activity

**DOI:** 10.1186/1479-5868-3-27

**Published:** 2006-09-14

**Authors:** Tom Baranowski

**Affiliations:** 1 Children's Nutrition Research Center, Department of Pediatrics, Baylor College of Medicine, 1100 Bates St., Houston, TX 77030, USA

## Abstract

Resnicow & Vaughn challenged the field of behavioral nutrition and physical activity to conduct research in new ways. They challenged the predictiveness of our models, sensitivity to initial conditions, factors predisposing to change and measurement procedures. While the predictiveness of our models will reflect the sophistication of our thinking and research, and the sensitivity to initial conditions is subsumed under the sophistication of our models, research on conditions predisposing to change (e.g. epiphanies), more longitudinal designs, refined measurement procedures and testing of critical issues can only enhance the quality of our research. Improved research quality should lead to enhanced efficacy and effectiveness of our interventions, and thereby our making meaningful contributions to mitigating the chaos in our field and the crisis from the rising epidemic of obesity.

## Background

**Figure 1:**
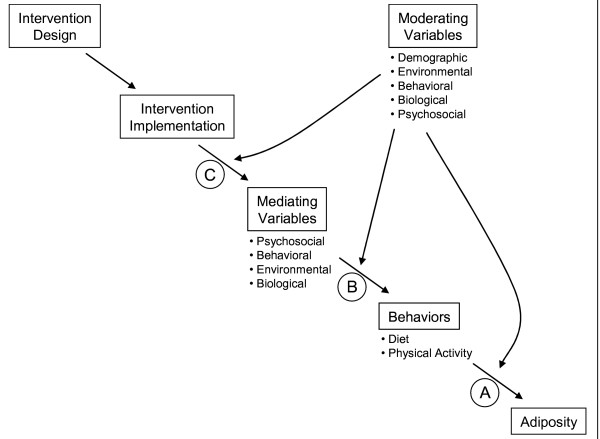
Mediating variable model for obesity.

Our field of behavioral nutrition and physical activity should be operating in crisis mode. The prevalence of obesity and overweight (an essentially nutrition and physical activity problem in its etiology and control) continues to increase at alarming rates in all age, demographic and gender groups in the US [1], Europe [2], and many other parts of the world [3,4]. There is concern that this will reverse the recent advances in chronic disease control [5]. In the face of this encroaching epidemic, obesity treatment programs have tended to have weak effects mostly for short periods of time [6]; and review after review have shown that obesity prevention programs also tend not to work [7-9]. Furthermore, using the mediating variable model (see Fig. 1) as a structured framework, it is not clear we know what changes in diet or physical activity behavior have led to the current problems and thereby provide the best behavioral targets for change [link A in Fig. 1] [10,11]; nor what mediating variables are most strongly related to these behaviors and thereby provide the best mechanisms for change [link B in Fig. 1] [12]; nor how best to manipulate the mediating variables to obtain behavior change and lower obesity [link C in Fig. 1] [13]. This is a frightful state of affairs. We should all be doing innovative theoretically guided, but high risk, research to quickly build a stronger knowledge base from which more effective interventions could be crafted. Yet, most of us appear to be acting in our usual way of doing things: "same old, same old,"

In this context, Resnicow & Vaughn [14] challenged our "same old" way of thinking about our field. They correctly specified the assumption of linearity in our predictive models, and proposed Chaos and Dynamic Systems Theories as alternative nonlinear models. They did not throw out all our theories per se, but challenged how we interrelated the variables, how we related them to behaviors, and offered some new variables predisposing to change. While Glass & McAtee [15] recently pointed out deficiencies in the social dimensions of our research, Resnicow & Vaughn targeted our thinking about behavior change. Some of the issues they raised are non-issues, but others deserve that we morph our basic methods to test the new ideas.

## Discussion

### Nonissues

Since we use statistical methods, all our models are by definition probabilistic, rather than deterministic (unless we could account for 100% of the variance, which will not happen in our lifetimes).

A key issue in their article was the predictiveness of our current models. They correctly identified the very limited predictiveness of the current models. The key issue, however, is the level of predictiveness that could possibly be achieved in predicting behavior. For example, our biological research colleagues are not satisfied unless their models account for 90% or more of the variance in their phenomena of interest. We are well below that [16]. Resnicow & Vaughn have not taken into account the emerging research on environmental influences, e.g. home availability [17], neighborhood characteristics [18,19]; biological influences, e.g. genes [20], sensitivity to tastes [21], the hormone rages of adolescent development [22,23]; emotional influences [23,24]; nor the likely interrelationships and interactions among these variables and our more usual psychosocial and behavioral predictors [25,26]. The higher the predictiveness of our models, the more we can engage in our logical approach to designing interventions based on these models. The larger number of and more diverse variables incorporated into these models, the more complex our interventions will need to be to address components of the model. And the interventions will need to both segment the population for differing types of interventions to different gender, age, ethnic, socioeconomic, and/or neighborhood groups, and tailor the intervention to individual characteristics within these groups [27]. At this time, we need to build and test the more comprehensive models. This is a daunting, but exciting, challenge.

Resnicow & Vaughn proposed the principal of sensitivity to initial conditions, as if this were a new idea. All of our models of longitudinal relationships (as equations) have built into them sensitivity to initial conditions, i.e. the initial values of the variables. How diverse the outcomes depends on the nature of the relationships. As our models become more comprehensive and complex, fairly similar initial conditions could lead to quite divergent outcomes. In part this is a function of the sophistication of our knowledge base. We need to build more sophisticated predictive models.

The idea of a tipping point or when it might occur, is not well defined [28]. In some ways it reifies a change, as if there is something intrinsic to or magical about the change process. If a tipping point is nothing more than a critical point on a variable beyond which change occurs, it is not clear the concept adds much, but identifying those points would be helpful.

### Issues deserving intensive research

Investigators could take away from the Resnicow & Vaughn message that change is random and cannot be predicted, and thereby cannot be understood by our usual research methods on behavior or its change. This would be very unfortunate. Resnicow & Vaughn will have made a major contribution, only if it leads to innovative research and new insights. Even in the vast complexities of molecular science, investigators are hammering away at delineating linear and nonlinear patterns to better understand the biology. Chances are we can do the same in behavior research.

Resnicow & Vaughn proposed that change does not occur in a linear "persuasion slowly overcoming resistance" manner, but rather in what they characterized as "quantum leaps," i.e. an epiphany or "aha!" event occurs from which the person decides to change. This is an interesting idea and should be testable. Innovative methods will be needed to identify people soon after the aha! experience to learn more about it. Perhaps interviewing new recruits to Weight Watchers™ or to fitness centers would accomplish this? A related issue would be what could we do to encourage aha! experiences? Are they a response to an overload of information (probably not, since we have done a lot of this already)? To repeated thinking about the issues (we could program prompts to thinking)? To setting off some emotional experience related to the behavior (we might be able to tailor messages to issues people found emotionally charged)? Resnicow & Vaughn invoked the concept of "cues" from the Health Belief Model. There has been some research on cues [29-31], but this has not as yet led to substantial insights. Relating cues to aha! experiences could be an important avenue for research. Developing valid and reliable retrospective methods to identify and recall aha! experiences would be necessary to make much progress.

Resnicow & Vaughn correctly pointed out the cross sectional nature of most of our research. Dynamic Systems modeling proposes that dynamic research be done, and this would be focused on change over time which requires longitudinal designs [32]. The importance of longitudinal designs was emphasized when Nigg [33] found that physical activity predicted ensuing self efficacy, but not the other way around. If self efficacy is really caused by physical activity, but doesn't cause physical activity, it doesn't make sense to try to increase self efficacy in interventions. While it is challenging to recruit and maintain longitudinal cohorts, such cohorts are required to address issues of direction of causality and thereby which variables should be targets for change in intervention programs. While ten year cohorts may not be necessary, perhaps 3 mo or 6 mo cohorts would provide tests for the changes we need. Longitudinal dynamic systems research has been initiated in other fields [34,35], which should provide a guide for our further development.

Whether behavior change can only be understood in retrospect instead of prospectively is an empirical issue. In part this is a function of how much variance our models will ultimately predict. Perhaps a few retrospective analyses will be necessary, perhaps using qualitative methods, to map out the processes occurring? But predictive science should be where we are headed, since predictive relationships clearly demonstrate what we know.

Resnicow & Vaughn correctly identified our current approaches to measurement as providing severe limitations to how we could understand our phenomena of interest. There have been limits on the extent to which existing measurement methods (e.g. classical test theory) have been used and reported [36], and limits on the predictiveness of existing measurement models [37]. One innovation in measurement theory that has recently drawn attention is Item Response Theory (IRT) [38]. IRT fits latent variables to items (and respondents) which identifies portions of the underlying variable being poorly measured [39], and assesses reliability across the range of the underlying variable [39,40]. Having items measuring specific locations on the underlying variable permits an assessment of whether the measures work differently after participation in an experiment [41]; differ by ethnic, gender or other groupings; and permit more efficient multidimensional modeling of the variable [42]. Use of IRT offers great promise for better understanding and minimizing the problems due to measurement of our constructs, and deserves much wider use.

An issue Resnicow addressed in his oral presentation in Boston (but not in his paper) was the falsifiability of a theory, and whether our current cognitive models are really theories. He correctly stated that in our current approach to research, no theories have been discarded (which would be considered a sign of progress and development in a field). Our best current research fits multivariate models to sets of variables [43], and determines which variables were significantly related to other variables in the model. This is useful for assessing the predictiveness of particular variables in certain situations, but does not necessarily address the usefulness of the larger theory. To move our field forward we need more attention to theoretical issues in our research, tests of clearer more specific predictions from theory applied to particular issues [44,45], and delineation of "critical issues" where two theories would make different predictions or model fitting research would need to test the fit of competing models [24], where the alternative models were predicated on different theories. More highly controlled experimental research on critical issues will also be necessary. Accumulation of findings across "critical" studies would enable the field to find more comprehensive and more predictive theoretical frameworks, and capitalize upon them in more likely to be effective interventions. There has been a distaste for theory in our field [46], and some have proposed continuing conducting intervention research until randomly hitting on intervention procedures that work [47]. Alternatively, I believe highly predictive theory should guide the design of effective interventions. In a complicated set of many possible variables and relationships, a random search may never result in finding effective change techniques, and even if it did, we wouldn't have the conceptual framework to understand why it happened in order to exploit it.

## Conclusion

Resnicow & Vaughn challenged the field of behavioral nutrition and physical activity to conduct research in new ways. While the predictiveness of our models will reflect the sophistication of our thinking and research, and the sensitivity to initial conditions is subsumed under the sophistication of our models, research on conditions predisposing to change (e.g. epiphanies), more longitudinal designs, refined measurement procedures and testing of critical issues can only enhance the quality of our research. Improved research quality should lead to enhanced efficacy and effectiveness of our interventions, and thereby our making meaningful contributions to mitigating the chaos in our field and the crisis from the rising epidemic of obesity.

## Abbreviations

US = United States

IRT = Item Response Theory

## Competing interests

The author(s) declare that they have no competing interests.
